# miR-148a-3p Mediates Notch Signaling to Promote the Differentiation and M1 Activation of Macrophages

**DOI:** 10.3389/fimmu.2017.01327

**Published:** 2017-10-16

**Authors:** Fei Huang, Jun-Long Zhao, Liang Wang, Chun-Chen Gao, Shi-Qian Liang, Dong-Jie An, Jian Bai, Yan Chen, Hua Han, Hong-Yan Qin

**Affiliations:** ^1^State Key Laboratory of Cancer Biology, Department of Medical Genetics and Developmental Biology, Fourth Military Medical University, Xi’an, China; ^2^Department of Stomatology, PLA Navy General Hospital, Beijing, China; ^3^Department of Oncology, Xijing Hospital, Fourth Military Medical University, Xi’an, China

**Keywords:** macrophages, Notch signaling, miR-148a-3p, PTEN, NF-κB

## Abstract

The Notch pathway plays critical roles in the differentiation and polarized activation of macrophages; however, the downstream molecular mechanisms underlying Notch activity in macrophages remain elusive. Our previous study has identified a group of microRNAs that mediate Notch signaling to regulate macrophage activation and tumor-associated macrophages (TAMs). In this study, we demonstrated that miR-148a-3p functions as a novel downstream molecule of Notch signaling to promote the differentiation of monocytes into macrophages in the presence of granulocyte macrophage colony-stimulating factor (GM-CSF). Meanwhile, miR-148a-3p promoted M1 and inhibited M2 polarization of macrophages upon Notch activation. Macrophages overexpressing miR-148a-3p exhibited enhanced ability to engulf and kill bacteria, which was mediated by excessive production of reactive oxygen species (ROS). Further studies using reporter assay and Western blotting identified *Pten* as a direct target gene of miR-148a-3p in macrophages. Macrophages overexpressing miR-148a-3p increased their ROS production through the PTEN/AKT pathway, likely to defend against bacterial invasion. Moreover, miR-148a-3p also enhanced M1 macrophage polarization and pro-inflammatory responses through PTEN/AKT-mediated upregulation of NF-κB signaling. In summary, our data establish a novel molecular mechanism by which Notch signaling promotes monocyte differentiation and M1 macrophage activation through miR-148a-3p, and suggest that miR-148a-3p-modified monocytes or macrophages are potential new tools for the treatment of inflammation-related diseases.

## Introduction

Macrophages have pivotal roles in development, homeostasis, and host defense. Ontogenically, macrophages originate from embryonic primitive and definitive hematopoiesis, as well as hematopoiesis in bone marrow (BM) in adults (1, 2). Hematopoietic stem cells in BM give rise to common myeloid progenitors, which produce monocytes through several intermediates, including macrophage and dendritic cell precursors and common monocyte progenitors. Monocytes emigrate from BM into the blood stream, both under steady state conditions, and on inflammatory activation (2).

In the periphery, macrophages participate in tissue surveillance and are activated by pathogens, tissue debris, and cytokines (3–5); however, the outputs of macrophage activation are heterogeneous, depending on signaling from pattern recognition receptors and the inflammatory microenvironment. Thus, lipopolysaccharide (LPS) and interferon γ (IFN-γ)-stimulated macrophages, or M1 macrophages, upregulate the expression of tumor necrosis factor (TNF)-α, interleukin (IL)-6, IL-12, and inducible nitric oxide synthase (iNOS, official symbol NOS2), accompanied by increased antigen presentation and bactericidal capacity. In contrast, IL-4-activated macrophages (M2 macrophages) express higher levels of immunosuppressive cytokines, such as IL-10 and transforming growth factor β, together with arginase-1, mannose receptor (MR; official symbol MRC1), and other molecules involved in anti-inflammation and tissue remodeling (3, 4). The multi-modality of macrophage activation has been implicated in many human diseases, including inflammatory diseases and cancer (5).

The Notch pathway is highly conserved through evolution and has a critical role in cell fate specification and development (6). Recombination signal-binding protein Jκ (RBP-J), a key transcription factor in Notch signaling, mediates signals from all four mammalian Notch receptors (Notch1–4) by binding with the activated Notch intracellular domain (NIC) (7). Notch signaling molecules are upregulated in activated macrophages (8–11), and Notch signaling is involved in several steps of macrophage differentiation and activation (12–19); however, the detailed molecular mechanisms, particularly downstream target(s), by which Notch signaling modulates macrophage differentiation and activation, remain elusive.

MicroRNAs (miRNAs) are a large class of non-coding small RNAs of approximately 21–23 nucleotides, which negatively regulate the expression of target genes through sequence-dependent recognition (20). Accumulating evidence indicates that various miRNAs participate in myeloid differentiation and macrophage activation (21–23). Previously, we compared miRNA expression profiles of BM-derived macrophages (BMDMs) from RBP-J-deficient and control mice, and demonstrated that miR-125a mediates Notch signaling to regulate M1/M2 macrophage polarization, and converts tumor-associated macrophages (TAMs) into M1-like macrophages to suppress tumor growth (18). In this study, we show that another candidate miRNA, miR-148a-3p, is a novel downstream molecule of Notch signaling in macrophage differentiation and activation. miR-148a-3p can promote osteoclastogenesis by targeting V-maf musculoaponeurotic fibrosarcoma oncogene homolog B, which is importantly involved in bone remodeling (24). We found that by targeting *Pten* to modulate the PTEN/AKT pathway, miR-148a-3p could not only promote M1 macrophage activation and inflammatory factor secretion through NF-κB but also enhance the ability of macrophages to engulf and kill bacteria *via* reactive oxygen species (ROS) production. These findings suggest that miR-148a-3p-mediated Notch signaling represents a novel mechanism in myeloid differentiation and macrophage activation, and is a potential therapeutic target for inflammation-related diseases.

## Materials and Methods

### Mice

Mice were maintained in a specific-pathogen-free facility on the C57BL/6 background. Lyz2-Cre mice (Stock #019096, The Jackson Laboratory) were crossed with RBP-J-floxed (RBP-J^f^) (25) or ROSA-Stop-floxed-NIC (STOP^f^-NIC, a gift from HL Li) mice to obtain Lyz2-Cre/RBP-J^+/f^ (Ctrl) and Lyz2-Cre/RBP-J^f/f^ (RBP-J^cKO^) mice, or Lyz2-Cre (Ctrl) and Lyz2-Cre/Stop^f^-NIC (NIC^cA^) mice (18). Mice were genotyped using PCR with mouse tail DNA as template. Primers are listed in Table S1 in Supplementary Material. All animal experiments were approved by the Animal Experiment Administration Committee of the Fourth Military Medical University and in accordance with the recommendations of *Guide for the Care and Use of Laboratory Animals* prepared by the National Academy of Sciences and published by the National Institutes of Health (NIH publication 86-23, revised 1985).

### Cell Culture and Transfection

Mouse BM cells were isolated from femurs and tibias. Nucleated cells were cultured in Dulbecco’s modified Eagle’s medium containing 10% fetal calf serum and 40 ng/mL murine granulocyte macrophage colony-stimulating factor (GM-CSF) (PeproTech, Rocky Hill, NJ, USA) for 7 days to obtain BMDMs. LPS (50 ng/mL, Sigma, St. Louis, MO, USA) and rIFN-γ (20 ng/mL, PeproTech) or IL-4 (20 ng/mL, PeproTech) were added into the BMDM culture for 24 h to induce M1 or M2 polarization, respectively. BMDMs were transfected with synthetic siRNA, miRNA mimics or inhibitors, and control oligoribonucleotides (RiboBio, Guangzhou, China) using Lipofectamine 2000™ (Invitrogen, Waltham, MA, USA) at a final concentration of 50 nmol/L, according to the manufacturer’s instructions. The sequence of miR-148a-3p mimic, inhibitor, and their controls was listed in Table S1 in Supplementary Material. The transfection efficiency of miRNA was determined in macrophages by labeling miR-148a-3p with Cy5 followed by FACS analysis.

The 3′ UTR of murine *Pten* was amplified using mouse tail DNA as a template and mutated by PCR-based methods using primers described in Table S1 in Supplementary Material. These fragments were inserted into the pGL3-promoter vector (Promega, Madison, WI, USA) to generate reporter plasmids. HeLa cells were seeded in 24-well plates and transfected with the reporter plasmids, with phRL-TK as an internal control. Cells were harvested 24 h after transfection, and relative luciferase activity was measured using the Dual Luciferase Reporter Assay System (Promega). Luciferase activity levels generated by the pGL3-promoter constructs were normalized to those of Renilla luciferase activity from phRL-TK.

### RNA Extraction and qRT-PCR

Total RNA was prepared from cultured macrophages using Trizol reagent (Invitrogen) according to the manufacturer’s instructions. Complementary DNA was synthesized using the PrimerScript RT Reagent Kit (Takara BIO, Dalian, China). Real-time quantitative PCR was performed using a SYBR Premix EX Taq kit (Takara, Japan) and run using the ABI PRISM 7500 Real-time PCR system (Life Technologies, Waltham, MA, USA), with β-actin and U6 RNA (for miRNAs) as internal controls. The primers used in PCR are shown in Table S1 in Supplementary Material.

### Western Blotting

BM-derived macrophages were harvested and lysed on ice for 30 min in RIPA buffer supplemented with protease inhibitors (Beyotime, Haimen, China), and lysates then centrifuged at 12,000 rpm for 10 min. The supernatants were collected, and protein concentrations were determined using BCA Protein Assay reagents (Pierce, Waltham, MA, USA). Nuclei were isolated using a kit (Beyotime) according to the supplier’s protocol and lysed as described above. Aliquots of cell lysates were separated by SDS-PAGE and then blotted onto PVDF membranes. Membranes were blocked with 5% skim milk solution for 2 h and then probed with primary antibodies, followed by horseradish peroxidase-conjugated goat anti-rabbit IgG (1:2,500) or goat anti-mouse IgG (1:2,000) (Boster Bio Tec, Wuhan, China). Protein blots were visualized using an ECL detection system (Pierce). The following primary antibodies were used: anti-PTEN, anti-Akt, anti-p-Akt, anti-IκB, anti-p65 and anti-Lamin A/C (Cell Signaling, Boston, MA, USA), anti-FIH1 (Abgent, San Diego, CA, USA), anti-HES1, and anti-β-actin (Sigma).

### Phagocytosis and Bacteria Killing Assay

BM-derived macrophages were seeded on coverslips in 24-well plates. *Escherichia coli* (BL21) (1 × 10^7^) carrying an EGFP-expressing plasmid were added to BMDMs at a 10:1 ratio (bacteria:BMDMs), followed by incubation at 37°C for 2 h. Free *E. coli* were removed by washing and the remaining cells were stained with biotin-anti-F4/80 antibody (eBioscience, San Diego, CA, USA), followed by Cy3-avidin (eBioscience). Samples were visualized under a fluorescence microscope (BX51, Olympus, Tokyo, Japan) or analyzed by flow cytometry, and the average number of engulfed EGFP^+^ *E. coli* per macrophage was calculated. To determine bacteria killing by macrophages, macrophages that had engulfed EGFP^+^ *E. coli* were incubated for a further 6 h. Cells were then lysed, diluted, and plated on ampicillin-containing agar plates. Bacterial colonies were counted and compared after overnight culture.

### Flow Cytometry

FACS analysis was performed using routine protocols with a FACS Calibur flow cytometer (BD Immunocytometry Systems, Franklin Lakes, NJ, USA). Antibodies for staining included APC anti-mouse CD11b (Biolegend, San Diego, CA, USA), Alexa 488 anti-mouse F4/80 (Biolegend), biotin anti-mouse Ly6G (Biolegend) and avidin-PE (eBioscience), PE anti-mouse MR (Biolegend). Dead cells were excluded by propidium iodide staining.

For intracellular staining, cells were stained with cell surface makers first, and then washed with PBS. After centrifuge, cell pellets were suspended completely with fixation buffer on ice for 30 min, and then permeabilized with a Permeabilization Buffer (eBioscience). After centrifuge again, cell pellets were washed and stained with PE anti-iNOS (eBioscience) followed by FACS analysis.

To determine ROS production, cells were harvested and labeled with a ROS probe, according to the recommended protocol (Beyotime), followed by FACS analysis. Briefly, the culture medium was removed and the cells were incubated in dark with the redox-sensitive fluorescent dye (DCFH-DA, 10 µM) diluted by serum-free medium at 37°C/5% CO_2_ incubator for 30 min. The cells were then washed with PBS and the fluorescent intensity of DCF was detected by FACS. The ROS level was analyzed using the mean fluorescent intensity (MFI).

### Mixed Lymphocyte Reaction

BM-derived macrophages were seeded in 24-well plates, treated with different stimuli for 24 h, and then irradiated with 20 Gy X-ray. Naïve T cells were negatively isolated from the lymph nodes of allogeneic mice using biotin-labeled B cell- and myeloid cells-specific antibodies, followed by magnetic avidin-microbeads (Miltenyi Biotec, Bergisch Gladbach, Germany). T cells were labeled with carboxyfluorescein diacetatesuccinimidyl ester and added to cultured BMDMs at a ratio of 5:1. Four days later, non-adherent cells were harvested and stained with APC anti-CD8 antibody (eBioscience). Proliferation of CD8^+^ T cells was analyzed by FACS.

### Enzyme-Linked Immunosorbent Assay (ELISA)

The supernatant of cultured BMDMs with different stimulation was used to measure the amount of TNF-α and IL-6 using ELISA Ready-SET-Go (eBioscience) according to the manufacturer’s instructions.

### Statistics

Western blotting images were imported into Image Pro Plus 5.1 software (MediaCybernetics Inc., Bethesda, MD, USA), and the density of electrophoretic bands were analyzed quantitatively. Data were analyzed with Graph Pad Prism 5 software (Version 5.0). Data were tested for normal distribution, and then unpaired student’s *t-*test, paired *t*-test or one way ANOVA with Turkey’s multiple comparison tests was used for statistical comparisons. *P* < 0.05 was considered statistically significant.

## Results

### Notch Signaling Promotes miR-148a-3p Expression in Macrophages

**Figure 1:**
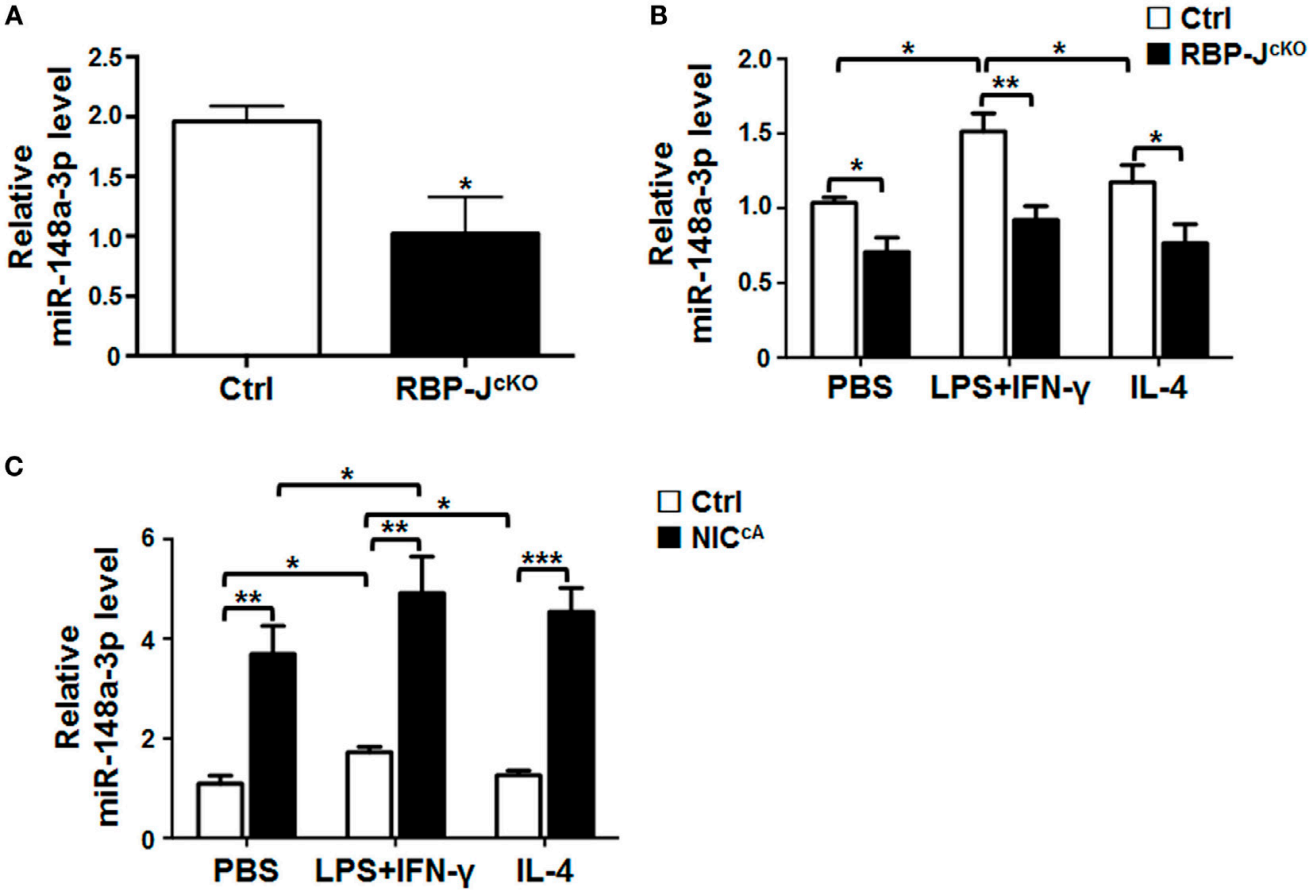
miR-148a-3p is downstream of Notch signaling in macrophages. **(A)** BM-derived macrophages (BMDMs) were cultured using bone marrow (BM) cells from RBP-J^cKO^ or control (Ctrl) mice. Levels of miR-148a-3p were determined by qRT-PCR, using U6 RNA as a reference control (*n* = 3). **(B)** BMDMs from RBP-J^cKO^ or control (Ctrl) mice were stimulated with PBS (M0), lipopolysaccharide (LPS) + interferon (IFN)-γ (M1), or interleukin (IL)-4 (M2). The level of miR-148a-3p was determined by qRT-PCR, using U6 RNA as a reference control (*n* = 6). **(C)** BMDMs were prepared using BM cells from NIC^cA^ or control (Ctrl) mice, and stimulated with PBS (M0), LPS + IFN-γ (M1), or IL-4 (M2). Levels of miR-148a-3p were determined by qRT-PCR, using U6 RNA as a reference control (*n* = 4). Bars, mean ± SD; *P < 0.05; **P < 0.01; ***P < 0.001.

Our previous miRNA profiling demonstrated that Notch signaling could regulate macrophage activation through a group of miRNAs (18). To assess whether miR-148a-3p could serve as a molecule downstream of Notch in macrophage regulation, we examined the expression of miR-148a-3p in macrophages with disrupted or ectopically activated Notch signaling, which is clarified by the expression level of HES1, one downstream molecule of Notch signaling (Figures S1A,B in Supplementary Material). The expression of miR-148a-3p was significantly downregulated in RBP-J-deficient BMDMs (Figure 1A). In BMDMs polarized with PBS (M0), LPS + IFN-γ (M1), or IL-4 (M2), RBP-J deficiency significantly reduced the expression of miR-148a-3p (Figure 1B). In contrast, when Notch signaling was constitutively activated by NIC overexpression, miR-148a-3p levels were dramatically increased in differently polarized macrophages (Figure 1C). These data suggest that Notch signaling can upregulate miR-148a-3p expression in unstimulated and activated macrophages.

### miR-148a-3p Promotes the Differentiation of Myeloid Cells into Macrophages

**Figure 2:**
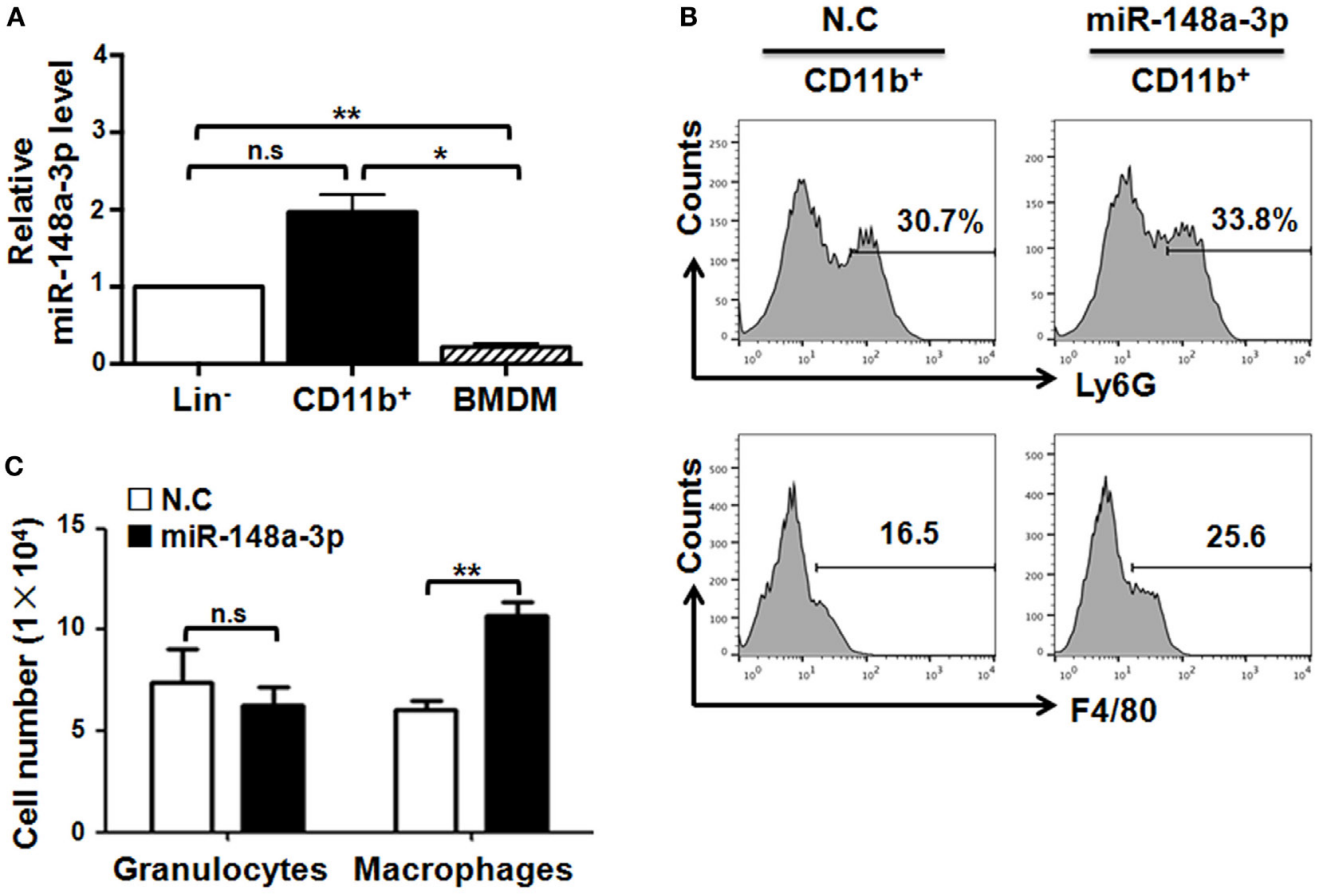
miR-148a-3p is involved in myeloid differentiation. **(A)** Bone marrow (BM) lineage^−^ (Lin^−^) and CD11b^+^ cells were sorted by MACS. Meanwhile, BM cells were cultured in the presence of granulocyte macrophage colony-stimulating factor (GM-CSF) for 7 days to obtain BM-derived macrophages (BMDMs). The relative level of miR-148a-3p was determined by qRT-PCR, using U6 RNA as a reference control (*n* = 3). **(B,C)** Monocytes were isolated from BM and transfected with miR-148a-3p mimic or a nonsense control (N.C.). Cells were then cultured in the presence of GM-CSF for 4 days, and subsequently analyzed by FACS **(B)**. Numbers of CD11b^+^Ly6G^+^ granulocytes and CD11b^+^F4/80^+^ macrophages were compared **(C)** (*n* = 3). Bars, mean ± SD; *P < 0.05; **P < 0.01; n.s., not significant.

Notch signaling promotes myeloid differentiation (26), and miR-148a-3p can participate in osteoclastogenesis (24). To assess the possibility that miR-148a-3p regulates macrophage differentiation, we sorted BM lineage^-^ (Lin^−^) hematopoietic stem and progenitor cells (HSPCs) and CD11b^+^ monocytes using MACS. Monocytes were further induced into mature BMDMs by culturing in the presence of GM-CSF for 7 days, and the level of miR-148a-3p was determined by qRT-PCR during macrophage differentiation. The results indicated that miR-148a-3p was highly expressed in monocytes, but presented at lower levels in HSPCs, with the lowest levels observed in BMDMs (Figure 2A). To further investigate the role of miR-148a-3p in myeloid development, miR-148a-3p mimic and its nonsense control (N.C.) were transfected into CD11b^+^ myeloid cells. After culturing with GM-CSF for 4 days, cells were analyzed by FACS. The results showed that the number of macrophages (CD11b^+^F4/80^+^) increased remarkably in myeloid cells overexpressing miR-148a-3p, while the number of granulocytes (CD11b^+^Ly6G^+^) did not change (Figures 2B,C). Taken together, these results indicate that miR-148a-3p could promote the differentiation of monocytes into macrophages.

### miR-148a-3p Can Promote M1 Macrophage Polarization

**Figure 3:**
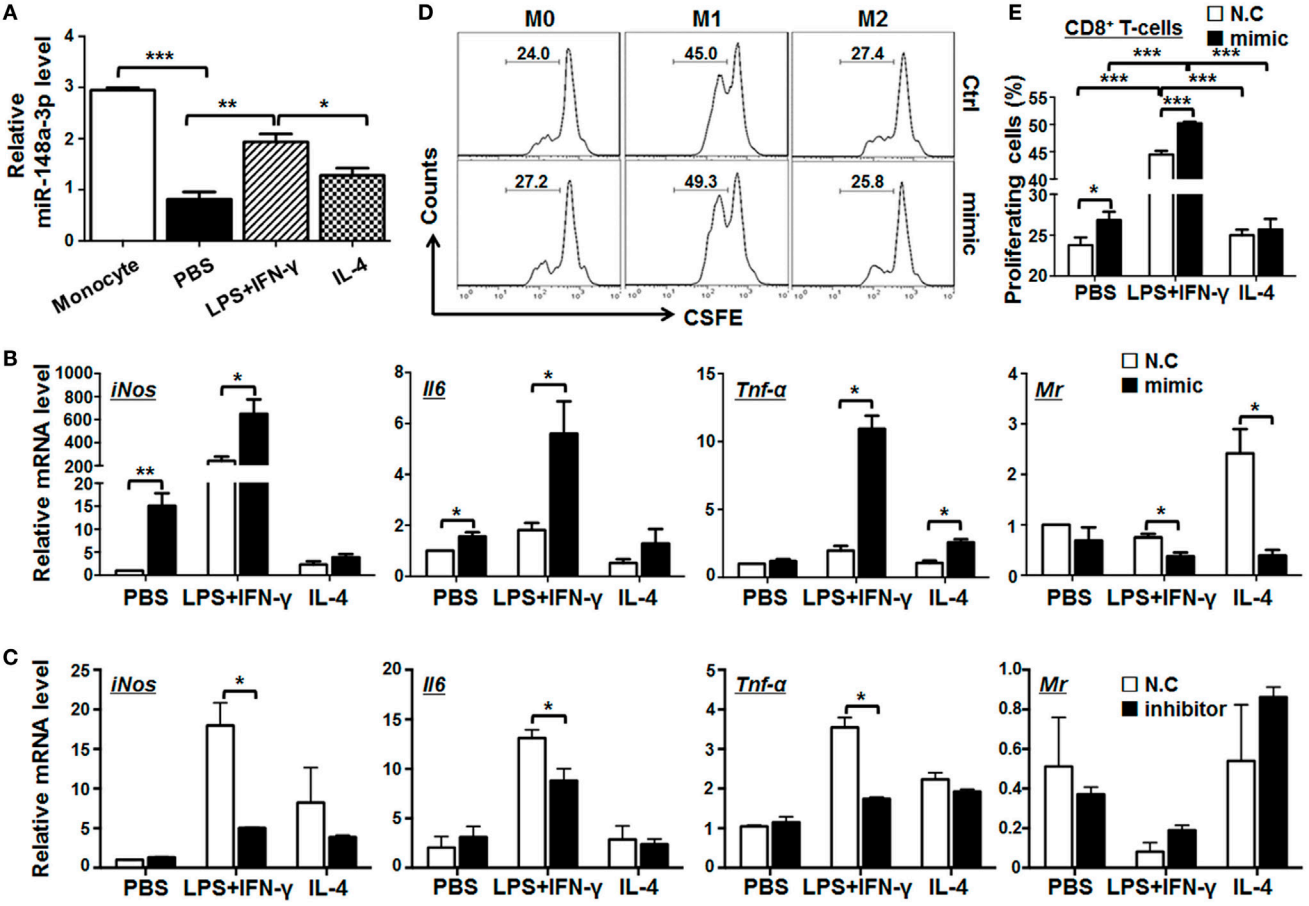
miR-148a-3p can promote M1 and inhibit M2 macrophage polarization. **(A)** BM-derived macrophages (BMDMs) were polarized with PBS (M0), lipopolysaccharide (LPS) + interferon (IFN)-γ (M1), or interleukin (IL)-4 (M2). The relative level of miR-148a-3p in monocytes and polarized BMDMs was determined by qRT-PCR, using U6 RNA as a reference control (*n* = 3). **(B)** BMDMs were transfected with miR-148a-3p mimic or N.C. Cells were then polarized with PBS (M0), LPS + IFN-γ (M1), or IL-4 (M2) for 24 h. The mRNA levels of *iNos, Il6, Tnf-*α, and *Mr* were determined by qRT-PCR, using β-actin (*ACTB*) as a reference control (*n* = 3). **(C)** BMDMs were transfected with miR-148a-3p inhibitor or N.C. Cells were then polarized with PBS (M0), LPS + IFN-γ (M1), or IL-4 (M2). The mRNA levels of *iNos, Il6, Tnf-*α, and *Mr* were determined by qRT-PCR, using β-actin as a reference control (*n* = 3). **(D,E)** BMDMs from **(A)** were irradiated and cocultured with carboxyfluorescein diacetatesuccinimidyl ester (CFSE)-labeled allogeneic T cells for 24 h. The proliferation of T cells was determined by FACS **(D)** and compared among treatment groups **(E)** (*n* = 6). Bars, mean ± SD; *P < 0.05; **P < 0.01; ***P < 0.001.

The expression of miR-148a-3p was upregulated in M1 macrophages polarized with LPS + IFN-γ, in comparison with that in M0 or M2 macrophages (Figures 1B and 3A). Next, mature BMDMs from normal mice were transfected with miR-148a-3p mimics or N.C. and stimulated with PBS (M0), LPS + IFN-γ, or IL-4 (M2) for 24 h. The transfection efficiency of miR-148a-3p in macrophages was almost 100% (Figure S2A in Supplementary Material). qRT-PCR demonstrated that the expression of the M1 markers, *iNos, Il6*, and *Tnf-*α increased, while that of the M2 marker *MR* decreased significantly in macrophages transfected with miR-148a-3p (Figure 3B). Consistently, the protein level of M1 markers including iNOS, IL-6, and TNF-α increased after miR-148a-3p transfection shown by FACS and ELISA (Figure S2B–G in Supplementary Material). Conversely, when BMDMs were transfected with a miR-148a-3p inhibitor, the expression of the M1 markers decreased clearly (Figure 3C). We cocultured allogenic naïve T cells with BMDMs that had been transfected with miR-148a-3p mimic and polarized with different stimuli. FACS analyses showed that unpolarized and LPS/IFN-γ-polarized BMDMs transfected with miR-148a-3p mimics exhibited significantly stronger ability to stimulate CD8^+^ T cell proliferation than that of control BMDMs (Figures 3D,E). These results indicate that miR-148a-3p can promote M1 polarization of macrophages.

### miR-148a-3p Enhances the Phagocytotic and Bactericidal Activities of Macrophages, Especially M1-Polarized Macrophages

**Figure 4:**
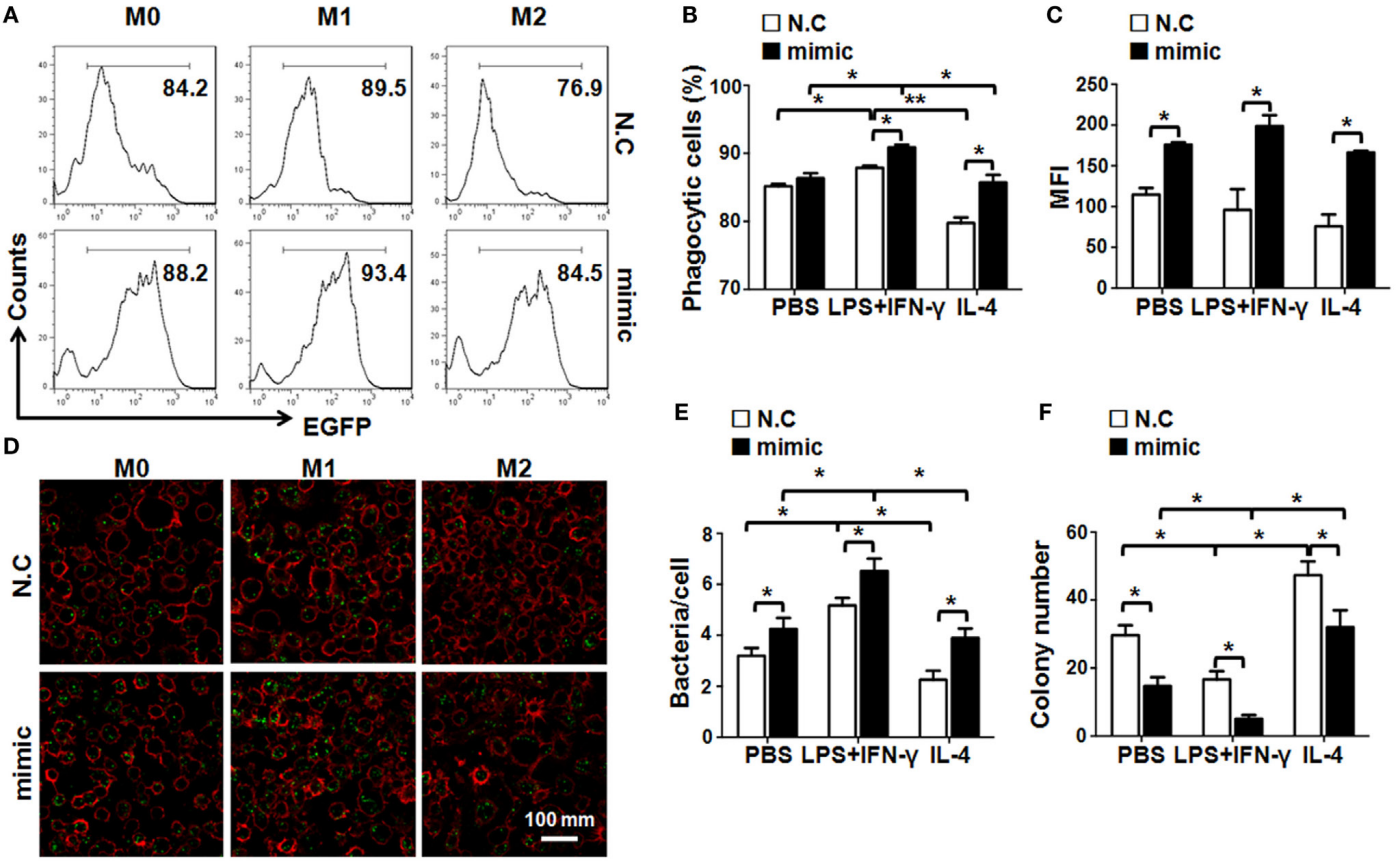
miR-148a-3p promotes macrophage phagocytosis and bactericidal activity. **(A)** BM-derived macrophages derived from normal mice were transfected with miR-148a-3p mimic or N.C. and stimulated with PBS (M0), lipopolysaccharide (LPS) + interferon (IFN)-γ (M1), or interleukin (IL)-4 (M2). Then, macrophages (1 × 10^6^) were cocultured with *E. coli* (1 × 10^7^) that had been transformed with an EGFP-expression vector for 2 h. Subsequently, the cells were washed and analyzed by FACS (*n* = 4). **(B)** The percentage of macrophages that had been engulfed by bacteria was compared between the miR-148a-3p mimic- and N.C.-transfected macrophages (*n* = 4). **(C)** The mean fluorescent intensity (MFI) of EGFP was compared between the miR-148a-3p mimic- and N.C.-transfected macrophages (*n* = 4). **(D,E)** Macrophages engulfing EGFP^+^ bacteria were photographed under a fluorescence microscope **(D)** and macrophages containing engulfed bacteria (green dots) were counted, and the numbers of bacteria per macrophage quantified and compared **(E)** (*n* = 3). **(F)** Macrophages that had engulfed EGFP^+^ *E. coli* were cultured for 6 h. Cells were then lysed and plated on ampicillin-containing agar plates. Bacterial colonies were counted and compared after overnight culture (*n* = 3). Bars, mean ± SD; *P < 0.05; **P < 0.01.

M1 macrophages exhibit stronger bactericidal activity (27). As miR-148a-3p enhanced M1 macrophage polarization, we next examined the phagocytosis and bactericidal activity of BMDMs transfected with miR-148a-3p mimic or control. BMDMs were transfected with miR-148a-3p or N.C. and activated, followed by incubation with EGFP^+^ *E. coli* for 2 h. FACS analyses showed that the percentage of EGFP^+^ macrophages increased significantly in the miR-148a-3p-transfected group (Figures 4A,B). Moreover, the MFI of EGFP in BMDMs transfected with miR-148a-3p also increased, suggesting that macrophages increased their levels of phagocytosis on transfection with miR-148a-3p, especially after M1 polarization (Figure 4C). This was confirmed by observation under a fluorescence microscope after anti-F4/80 staining, which demonstrated that the number of bacteria per cell increased in miR-148a-3p-transfected BMDMs (Figures 4D,E). To determine the bactericidal ability of macrophages, we cocultured differentially polarized macrophages with *E. coli* for 6 h, and determined the number of live intracellular bacteria by plating on Amp^+^ agar plates. Numbers of surviving bacteria were reduced in miR-148a-3p-transfected BMDMs compared with the control, particularly in M1 macrophages (Figure 4F; Figure S3 in Supplementary Material). These results indicate that miR-148a-3p can enhance the phagocytotic and bactericidal activity of macrophages, especially M1-polarized macrophages.

### *Pten* Is a Direct Target of miR-148-3p in Macrophages

**Figure 5:**
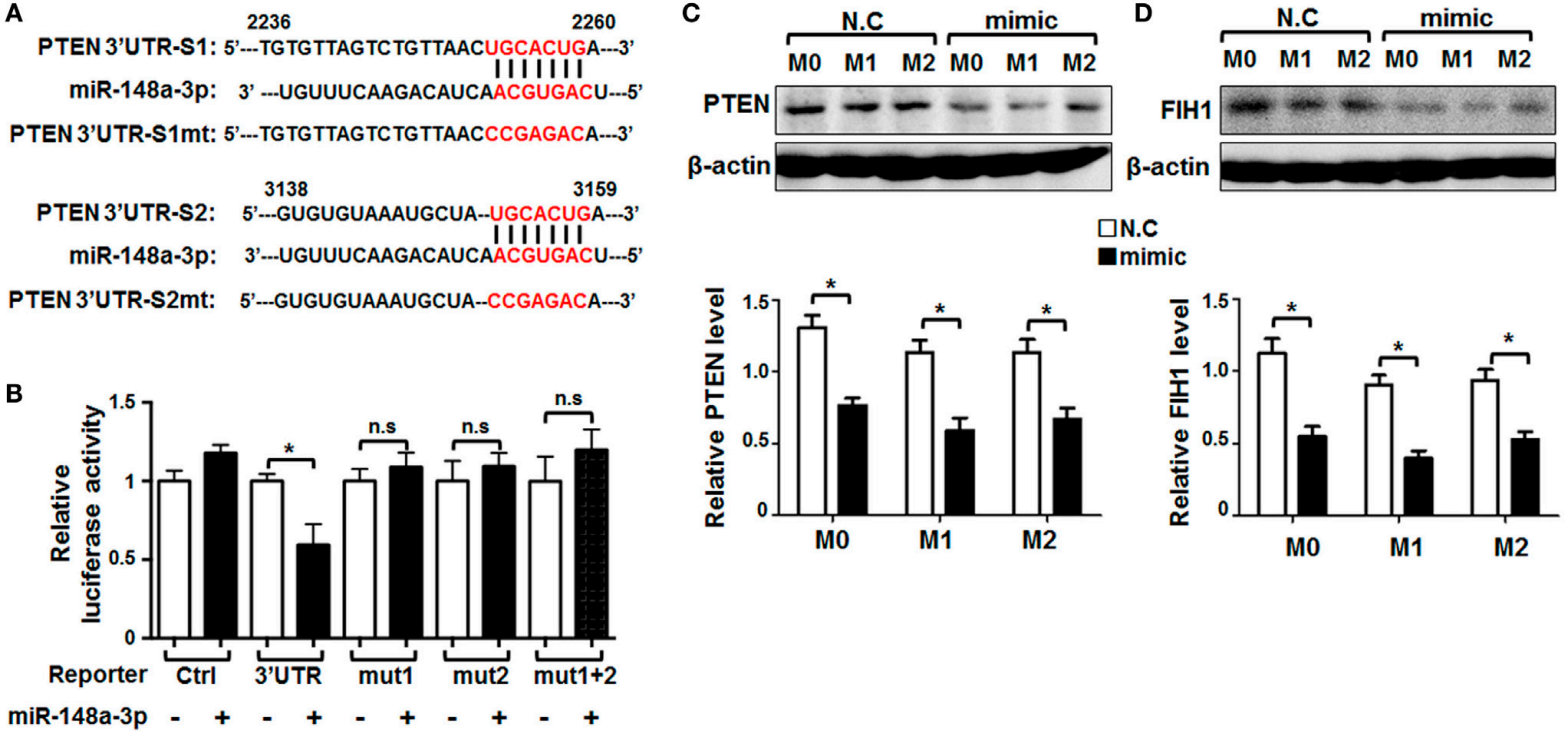
Pten is a target of miR-148a-3p in macrophages. **(A)** The sequence of the 3′-UTR of *Pten* was aligned with the seed sequence of miR-148a-3p. The potentially recognized sequences (nucleotides 2,253–2,259 and 3,152–3,158) are highlighted in red. The sequences of the mutated *Pten* 3′-UTR are also shown. **(B)** HeLa cells were transfected with miR-148a-3p mimic or N.C., together with reporters containing wild-type and mutant *Pten* 3′-UTRs. Luciferase activity was determined 24 h after transfection (*n* = 6). **(C,D)** BM-derived macrophages were transfected with miR-148a-3p mimic or N.C., and then polarized with PBS (M0), LPS + IFN-γ (M1), or IL-4 (M2). PTEN and factor inhibiting hypoxia-inducible factor 1 (FIH1) protein levels were determined by Western blotting, using β-actin as a reference control, and then, the relative levels of PTEN **(C)** and FIH1 **(D)** was compared quantitatively (*n* = 3). Bars, mean ± SD; *P < 0.05; n.s, not significant.

Recently, there have been several reports that *Pten* is a target of miR-148a in inflammation-related diseases (28–30). To determine whether *Pten* is also a key target of miR-148a directly involved in macrophage activation, we predicted targets of miR-148a-3p using several bioinformatic algorithms, including Target Scan, PicTar, and miRDB, and identified two conserved motifs in the *Pten* 3′-UTR that are potentially recognized by the miR-148a-3p seed sequence (Figure 5A). We then constructed luciferase reporter genes using wild-type *Pten* 3′-UTR or mutated derivatives, including mut1 (with the predicted binding site at 2,253–2,259 bp disrupted), mut2 (with the predicted binding site at 3,152–3,158 bp disrupted), and mut1 + mut2 (with both predicted binding sites disrupted) (Figure 5A). HeLa cells were transfected with each of the different reporters and miR-148a-3p. Subsequent luciferase reporter assays indicated that miR-148a-3p indeed repressed luciferase activity regulated by the wild-type *Pten* 3′-UTR (Figure 5B); however, disruption of either or both of the predicted miR-148a-3p binding sites in the *Pten*3′-UTR abrogated the repression activity of miR-148a-3p (Figure 5B), suggesting both sites are required simultaneously for miR-148a-3p activity.

In addition, BMDMs were transfected with miR-148a-3p or N.C. and polarized. PTEN protein levels were determined by Western blotting, as well as factor inhibiting hypoxia-inducible factor 1(FIH1)protein levels as known target of miR-148a-3p were detected (31). The results showed that PTEN and FIH1 protein levels were significantly reduced by miR-148a-3p in unstimulated or M1-polarized BMDMs (Figures 5C,D). These results suggest that miR-148a-3p can repress PTEN and FIH1 expression in macrophages.

### miR-148a-3p Can Promote M1 Macrophage Polarization *via* PTEN/AKT/NF-κB Signaling

**Figure 6:**
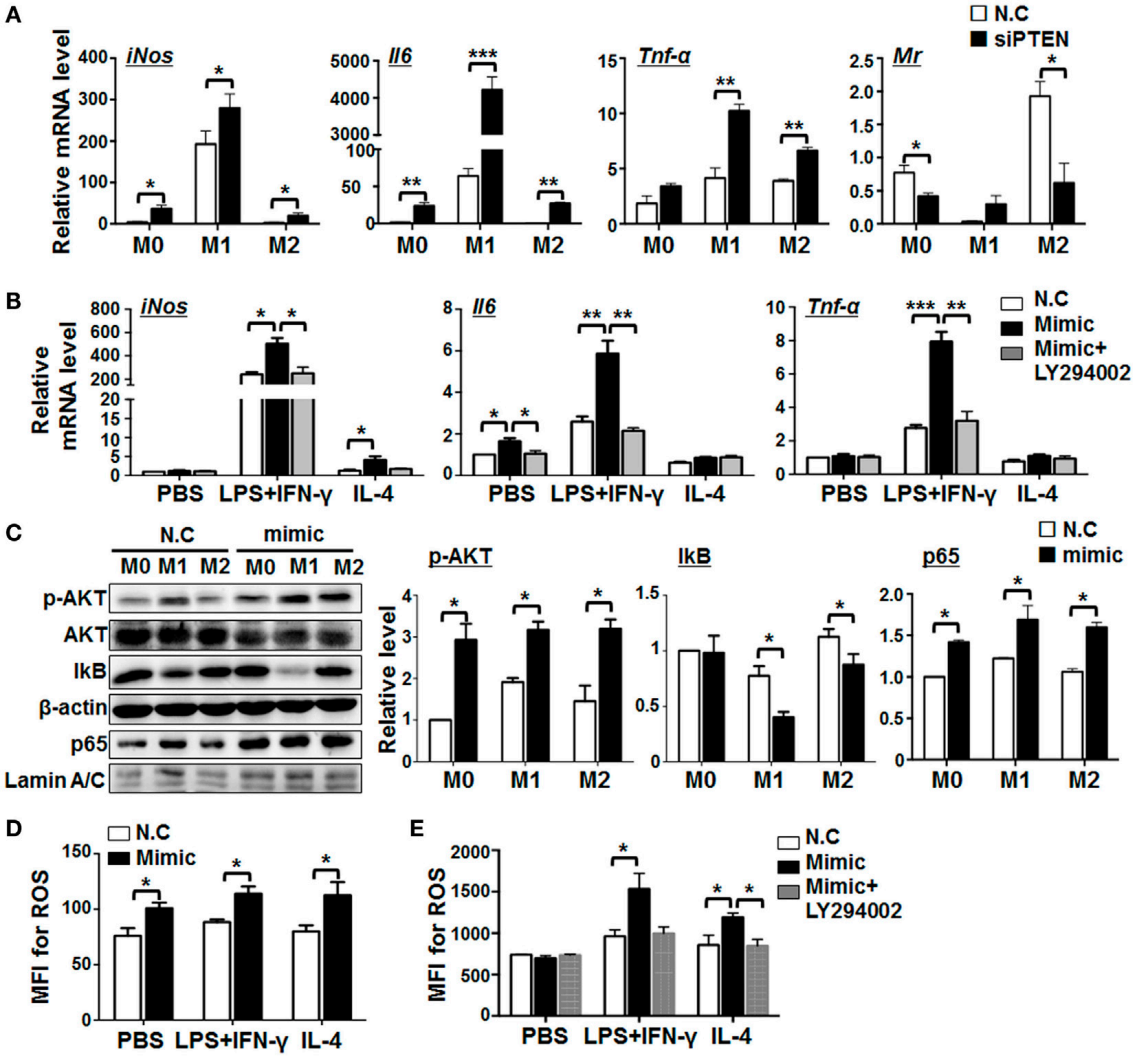
miR-148a-3p promotes M1 macrophage polarization *via* PTEN/AKT/NF-κB signaling. **(A)** BM-derived macrophages (BMDMs) were transfected with siRNA targeting *Pten* (siPTEN) or N.C., and then polarized with PBS (M0), lipopolysaccharide (LPS) + interferon (IFN)-γ (M1), or interleukin (IL)-4 (M2). mRNA levels of *iNos, Il6, Tnf-*α, and *Mr* were determined by qRT-PCR, using β-actin as a reference control (*n* = 3). **(B)** BMDMs were transfected with miR-148a-3p mimic or N.C., and then polarized with PBS (M0), LPS + IFN-γ (M1), or IL-4 (M2), in the presence of the AKT inhibitor, LY294002. The mRNA levels of *iNos, Il6*, and *Tnf-*α were determined by qRT-PCR, using β-actin as a reference control (*n* = 3). **(C)** BMDMs were transfected with miR-148a-3p mimic or N.C., and then polarized with PBS (M0), LPS + IFN-γ (M1), or IL-4 (M2). The levels of phosphorylated AKT (p-AKT), total AKT, IκB, and nuclear p65 proteins were determined by Western blotting, using β-actin or Lamin A/C as reference controls, and quantitatively compared (*n* = 3). **(D)** BMDMs were transfected with miR-148a-3p mimic or N.C., and then polarized with PBS (M0), LPS + IFN-γ (M1), or IL-4 (M2). Reactive oxygen species (ROS) levels were determined using FACS (Figure S4A in Supplementary Material) and expressed as mean fluorescent intensity (MFI) (*n* = 3). **(E)** BMDMs were transfected with miR-148a-3p mimic or N.C., and then polarized with PBS (M0), LPS + IFN-γ (M1), or IL-4 (M2), in the presence of LY294002. ROS levels were determined using FACS (Figure S4B in Supplementary Material) and are expressed as MFI (*n* = 3). Bars, mean ± SD; *P < 0.05; **P < 0.01; ***P < 0.001.

Next, we assessed the role of PTEN in miR-148-3p-mediated macrophage activation. BMDMs were transfected with siRNA specifically targeting *Pten* (siPTEN) followed by different polarization treatments. The results demonstrated that knockdown of PTEN led to upregulation of the M1 markers *iNos, Il6*, and *Tnf-*α while the M2 marker, *Mr*, was downregulated (Figure 6A). To further determine whether miR-148a-3p can promote M1 macrophage polarization through PTEN/AKT signaling, BMDMs were transfected with miR-148a-3p and stimulated with different polarizing agents in the presence of the AKT inhibitor, LY294002. Subsequent qRT-PCR demonstrated that, although miR-148a-3p upregulated the M1 markers *iNos, Il6*, and *Tnf-*α, this effect was prevented by treatment with the AKT inhibitor, LY294002 (Figure 6B). These data suggest that miR-148a-3p can promote M1 macrophage polarization by repressing PTEN/AKT signaling.

The PTEN/PI3K/AKT pathway is critically involved in multiple cellular events including proliferation, survival, and apoptosis (32, 33). NF-κB signaling is a target of PI3K/AKT signaling (34) and downregulation of PTEN activates NF-κB signaling by promoting nuclear translocation of p65 in mouse mesangial cells (35). Moreover, AKT and/or NF-κB pathway have been extensively implicated in M1 polarization of macrophages (36). Thus, we hypothesized that miR-148a-3p might regulate M1 macrophage polarization *via* PTEN/AKT/NF-κB signaling. Therefore, BMDMs were transfected with miR-148a-3p mimics or N.C., followed by different polarization treatments, and the levels of AKT, phosphorylated AKT, IκB, and nuclear p65 were determined by Western blotting, using β-actin or Lamin A/C as internal controls. The results demonstrated that miR-148a-3p can significantly promote AKT phosphorylation in macrophages. Meanwhile, levels of IκB were reduced, whereas those of nuclear p65 increased (Figure 6C). Therefore, miR-148a-3p can promote M1 macrophage polarization *via* the PTEN/AKT/NF-κB signaling pathway.

miR-148a-3p enhanced the bactericidal activity of macrophages (Figure 4), likely through the production of ROS (37). Next, we determined levels of ROS production in differentially polarized BMDMs transfected with miR-148a-3p mimic or N.C. by FACS analysis. The results showed that miR-148-3p enhanced ROS production by BMDMs (Figure 6D and Figure S4A in Supplementary Material), and treatment with the AKT inhibitor, LY294002, abolished this effect (Figure 6E; Figure S4B in Supplementary Material). These results indicate that miR-148a-3p can enhance the ability of BMDMs to kill bacteria by increasing ROS production through the PTEN/AKT pathway.

### Notch Signaling Promotes Inflammatory Factors Secretion and ROS Production in M1 Macrophages *via* miR-148a-3p

**Figure 7:**
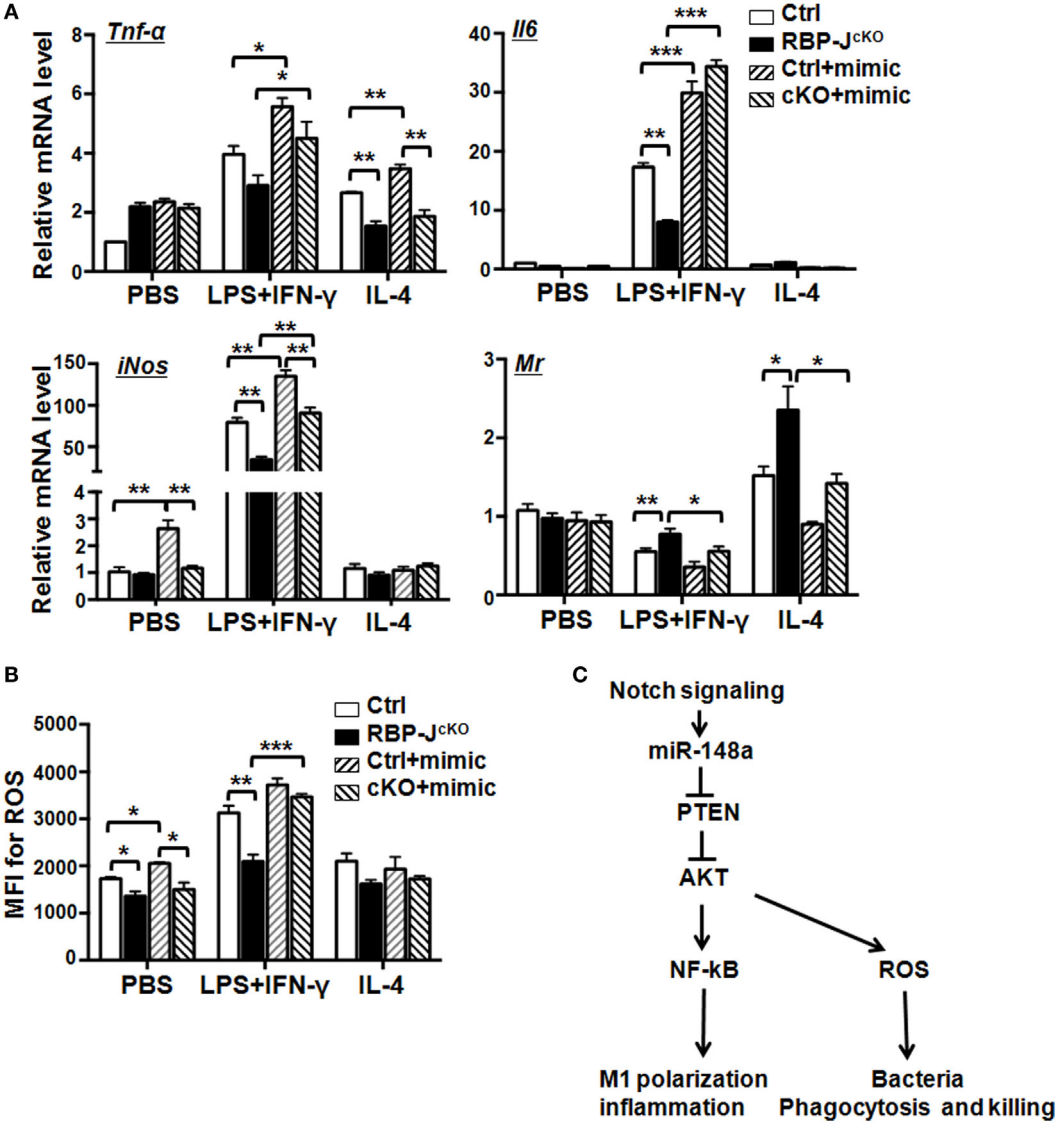
Notch signaling promotes secretion of inflammatory factors and reactive oxygen species (ROS) production of M1 macrophage *via* miR-148a-3p. **(A)** BM-derived macrophages (BMDMs) were cultured using bone marrow cells from RBP-J^cKO^ (cKO) or control (Ctrl) mice. Cells were transfected with miR-148a-3p mimic or N.C., and then polarized with PBS (M0), LPS + interferon (IFN)-γ (M1), or interleukin (IL)-4 (M2). The mRNA levels of *Tnf-*α, *Il6, iNos*, and *Mr* were detected by qRT-PCR (*n* = 3). **(B)** BMDMs were treated as described in panel **(A)**. ROS levels were determined by FACS and expressed as mean fluorescent intensity (MFI) (*n* = 3). **(C)** Schematic illustrating how Notch signaling can enhance M1 macrophage polarization and bacterial killing through miR-148a-3p (see details in the text). Bars, mean ± SD (*n* = 6); *P < 0.05; **P < 0.01.

Notch activation promoted M1, but inhibited M2, macrophage polarization (Figure S5A in Supplementary Material), while RBP-J-deficiency produced the opposite effects on macrophages (Figure S5B in Supplementary Material). The production of ROS increased in macrophages on Notch activation, but decreased in RBP-J-deficient macrophages (Figures S5C,D in Supplementary Material). To investigate whether miR-148a-3p functions downstream of Notch signaling to regulate macrophage polarization, we cultured BMDMs from RBP-J KO and control mice and transfected them with miR-148a-3p mimic or N.C., followed by polarization with PBS (M0), LPS + IFN-γ (M1), or IL-4 (M2). The expression of inflammatory factors and activated macrophage markers were then evaluated by qRT-PCR. The results demonstrated that RBP-J disruption reduced the expression of *Tnf-*α, *Il6*, and *iNos* on LPS + IFN-γ stimulation, whereas the expression of *Mr* was enhanced on IL-4 stimulation, and that these changes were reversed by transfection with miR-148a-3p mimic (Figure 7A). Moreover, RBP-J blockade reduced ROS production in M1 macrophages, and miR-148-3p restored this effect (Figure 7B). Taken together, these data indicate that miR-148a-3p mediates Notch signaling to enhance the production of inflammatory factors and ROS in M1 macrophages, which may contribute to defense against pathogenic invasion.

## Discussion

The Notch pathway determines a large number of cell fate specification events during embryonic and postnatal development through mediation of juxtacrine signaling. In recent years, Notch signaling has also been implicated in the regulation of cell plasticity under various physiological and pathological conditions and contributes broadly to the establishment and maintenance of the immune system (38). Several groups, including ours, have reported that Notch signaling regulates the development of myeloid cells and the functional heterogeneity of macrophages (12–19). Our previous study demonstrated that activated Notch signaling is required for M1 macrophage polarization. In contrast, RBP-J-deficient macrophages incline toward M2 polarization and possess attenuated antitumor function, even after LPS + IFN-γ stimulation (13). To identify downstream molecules mediating Notch signaling in regulation of macrophage development and activation, we compared miRNA profiles of Notch-inactivated and control macrophages, and found that miR-148a-3p showed significantly decreased expression in RBP-J-deficient macrophages (18). miR-148a-3p has been reported to promote osteoclastogenesis (24). In this study, we further validated miR-148a-3p as a novel downstream molecule of Notch signaling, which can modulate myeloid differentiation and promote M1 macrophage activation. Han et al. reported that conditional RBP-J deletion in BM does not significantly alter the number of myeloid colony-forming units (25), suggesting that the effects of Notch signaling on myeloid development could be stage- and context-dependent. Indeed, Notch signaling has been implicated in various myeloid malignancies (39) and disruption of Notch signaling leads to myeloproliferative disorders in mice (22, 40). Our current study demonstrates that overexpression of miR-148a-3p in BM cells promotes monocyte to macrophage differentiation, but has no effect on granulocyte development in the presence of GM-CSF. From our data and the literature, we conclude that a set of miRNAs, including miR-125a (18), miR-155 (22), and miR-148a-3p (this study), mediate Notch signaling in regulation of macrophage development. More detailed studies are required to elucidate how Notch signaling regulates these miRNAs cooperatively and what targets and related signaling pathways are influenced by these miRNAs in macrophages.

miR-148a-3p can promote M1 macrophage polarization to regulate inflammation. A growing body of research demonstrates that miRNAs are regulators of the inflammatory responses of immune cells, including macrophages (21). In this study, we demonstrated that *Pten* is a direct target of miR-148a-3p in activated macrophages. Reduction of PTEN levels leads to increased activation of AKT, which in turn results in enhanced NF-κB activation followed by M1 macrophage polarization and secretion of inflammatory factors (Figure 7C). Our results further confirmed the previous study of AKT and NF-κB pathway participating in M1 macrophage polarization (36). PTEN is a tumor suppressor, and the mutation or deletion of PTEN results in multiple human diseases, including inflammation-related disorders and cancer (28, 41, 42). Multiple miRNAs have been reported to target *Pten*; for example, miR-21 contributes to hepatocellular carcinoma (HCC) through inhibiting PTEN expression (43). miR-32 also affects the proliferation, migration, and invasion of HCC cells *via* the PTEN/AKT signaling pathway (44). In ovarian cancer, miR-214 induces cell survival and cisplatin resistance, primarily through targeting the PTEN/AKT pathway (45). In addition, miR-26a-mediated PTEN repression is amplified in high-grade glioma and facilitates gliomagenesis *in vivo* (46). Zhang et al. showed recently that miR-26b participates in the inflammatory response of bovine alveolar macrophages by modulating NF-κB signaling through targeting *Pten* (47). In particular, several recent papers have reported that miR-148a-3p-mediated inhibition of *Pten* participates in glomerular cell proliferation in lupus nephritis (28), osteosarcoma cell growth (29), and immature B cells survival (30), suggesting that miR-148a-3p can regulate inflammation-related diseases by targeting *Pten*. Given the important role of macrophages during inflammation initiation and progression, the results of the current study show that miR-148a-3p can promote inflammatory responses of LPS/IFN-γ-stimulated M1 macrophages by modulating PTEN/AKT/NF-κB signaling. These findings suggest that expression levels of *Pten* can be regulated by a set of miRNAs, and indicate that targeting *Pten* through epigenetic modification may be a useful therapeutic strategy for patients suffering from inflammation-related diseases and cancer.

Recently, Wong et al. has reported that FIH1 as a direct target of miR-148a-3p regulates stem cells and angiogenesis in glioblastoma through HIF1α and Notch pathway (31). Because FIH1 is expressed in macrophages (18), in this study, we clarified that the expression of FIH1 in different polarized macrophages is reduced after miR-148a-3p overexpression (Figure 5D), suggesting that FIH1 could be another target of miR-148a-3p in macrophages. However, whether miR-148a-FIH1/HIF1α can participate in M1 macrophages activation and inflammation response through Notch signaling needs further exploring.

In this study, we also confirmed that overexpression of miR-148a-3p in macrophages enhanced ROS production, which likely caused the increased bactericidal activity of macrophages. miR-148a-3p promotes ROS production in M1 macrophages through the PTEN/AKT pathway (Figure 7C). As ROS not only activates PI3K/AKT signaling but also concurrently inactivates PTEN (37); it is reasonable to assume that miR-148a-3p may modulate the M1 macrophage phenotype through a positive feedback loop between ROS and PTEN/AKT signaling, ultimately leading to activated NF-κB signaling and increased release of pro-inflammatory factors to support host defense against invasive microbial pathogens. However, although appropriate inflammatory responses may protect hosts from exogenous and endogenous insults, unnecessarily excessive inflammation can cause host tissue damage. To maintain tissue homeostasis and optimize host responses, specific molecular networks are required to control the initiation and resolution of inflammation (48). Recent studies suggest that ROS can not only activate NF-κB but also has the potential to repress NF-κB activity (49). Whether Notch signaling-mediated ROS release in LPS/IFN-γ-stimulated M1 macrophages through miR-148a-3p/PTEN/AKT signaling can also repress excessive inflammatory responses induced by NF-κB signaling, requires further elucidation.

Macrophages have been regarded as potential therapeutic targets for a variety of chronic inflammatory diseases and cancer; however, these cells display remarkable heterogeneity in their ontogeny and activation and, therefore, exert multi-modal roles in regulating inflammatory responses (1–5). How to program and/or reprogram macrophages from destructive to beneficial phenotypes in disease therapy remains a challenge. Our previous work has shown that Notch signaling can participate in reprogramming of TAMs to M1-like macrophages with antitumor activity through miR-125a (18). Baer et al. reported that depletion of the miRNA-processing enzyme, DICER, in macrophages promotes M1-like TAM programming through hyperactivation of IFN-γ/STAT1 signaling (23). Recently, Guerriero et al. showed that the class IIa histone deacetylase inhibitor, TMP195, can harness the antitumor potential of macrophages to enhance cancer therapy (50). These studies raise the possibility of a promising therapeutic strategy of applying epigenetic tools to reprogram the phenotype of macrophages. In this study, we demonstrate that miR-148a-3p-mediated Notch signaling promotes M1 macrophage polarization and the bactericidal ability of macrophages. Whether miRNAs- or Notch-modified macrophages can be used as a novel therapeutic strategy for inflammation-related diseases, will require additional research.

In conclusion, the current study demonstrates, for the first time, that miR-148a-3p participates in myeloid cell differentiation. Moreover, miR-148-3p is induced in activated macrophages in a Notch-dependent manner, which both promotes an inflammatory response through NF-κB and enhances phagocytosis and bactericidal activity through ROS release, by targeting the PTEN/AKT pathway (Figure 7C).

## Ethics Statement

This study was carried out in accordance with the recommendations of Guide for the Care and Use of Laboratory Animals prepared by the National Academy of Sciences and published by the National Institutes of Health (NIH publication 86-23, revised 1985). The protocol was approved by the Animal Experiment Administration Committee of the Fourth Military Medical University.

## Author Contributions

FH, J-LZ, and LW performed experiments, analyzed data, wrote the draft, and reviewed the manuscript. C-CG, S-QL, D-JA, JB, and YC performed some of the experiments, analyzed data, and reviewed the manuscript. HH and H-YQ designed the project, analyzed and integrated data, and wrote the manuscript.

## Conflict of Interest Statement

The authors declare that the research was conducted in the absence of any commercial or financial relationships that could be construed as a potential conflict of interest.

## Abbreviations

BM, bone marrow; BMDMs, BM-derived macrophages; iNOS, inducible nitric oxide synthase; FIH1, factor inhibiting hypoxia-inducible factor 1; MR, mannose receptor; ROS, reactive oxygen species; RBP-J, recombination signal-binding protein Jκ; NIC, Notch intracellular domain; TAMs, tumor-associated macrophages.
