# Supplementary material for: miR-148a-3p Mediates Notch Signaling to Promote the Differentiation and M1 Activation of Macrophages
Source: Front Immunol. 2017 Oct 16;8:1327. doi: 10.3389/fimmu.2017.01327 (PMC5650608; doi:10.3389/fimmu.2017.01327)
Supplement: Supplementary file 1 [file Data_Sheet_1.pdf]

Supplementary Material

miR-148a-3p Mediates Notch Signaling to Promote the Differentiation and M1 Activation of Macrophages

Fei Huang, Jun-Long Zhao, Liang Wang, Chun-Chen Gao, Shi-Qian Liang, Dong-Jie An, Jian Bai, Yan Chen, Hua Han*, Hong-Yan Qin*

*** Correspondence:** Hua Han: huahan@fmmu.edu.cn; Hong-Yan Qin: hyqin@fmmu.edu.cn

Table S1. Primers and oligonucleotides used for PCR in this study.

Name	Purpose	Sequence
Cre-F*	Genotyping	5'-CCGGTCGATGCAACGAGTGATGAGG
Cre-R*	Genotyping	5'-GCCTCCAGCTTGCATGATCTCCGG
RBP-J-F	Genotyping	5'-GTTCTTAACCTGTTGGTCGGAACC
RBP-J-WT-R	Genotyping	5'-GCTTGAGGCTTGATGTTCTGTATTGC
RBP-J-floxed-R	Genotyping	5'-ACCGGTGGATGTGGAATGTGT
NICD-F	Genotyping	5'-AAAGTCGCTCTGAGTTGTTAT
NICD-WT-R	Genotyping	5'-TAAGCCTGCCCAGAAGACTC
NICD-floxed-R	Genotyping	5'-GAAAGACCGCGAAGAGTTTG
PTEN-F	RT-PCR	5'-ATCAAGAGGATGGATTCG
PTEN-R	RT-PCR	5'-GGCGGTGTCATAATGTCT
PTEN mut1-F	PCR	5'-CAATGGGCTGTCCGAGACTTAATA
PTEN mut1-R	PCR	5'-TATTAAGTCTCGGACAGCCCATTG
PTEN-mut2-F	PCR	5'-AAATGCTACCGAGACAGGATACAC
PTEN-mut2-R	PCR	5'-GTGTATCCTGTCTCGGTAGCATTT
PTEN 3'UTR-F	PCR	5'-CGGAATTCGAAATCTGTACACCCCCTTGTCTT
PTEN 3'UTR-R	PCR	5'-AACTGCAGCACCCACACAATGACAAGAATGAG
PTEN siRNA	siRNA	5'-TCTTCAAGGGCAATTTGCTCATTAA
iNOS-F	RT-PCR	5'-TCGACATCCGCAACGACTATC
iNOS-R	RT-PCR	5'-CCAGGGCGTAGTTGTAGAAGAG
IL-6-F	RT-PCR	5'-ATGGCCCATTACAAAGCCG
IL-6-R	RT-PCR	5'-TTTCTGGAGTAGCAGCTCCTAA
TNF-α-F	RT-PCR	5'-TCGACATCCGCAACGACTATC
TNF-α-R	RT-PCR	5'-CCAGGGCGTAGTTGTAGAAGAG
MR-F	RT-PCR	5'- AAACACAGACTGACCCTTCCC
MR-R	RT-PCR	5'- GTTAGTGTACCGCACCCTCC
β-actin	RT-PCR	5'-CATCCGTAAAGACCTCTATGCCAAC
β-actin	RT-PCR	5'-ATGGAGCCACCGATCCACA
control mimics	nucleotides	5'-UUUGUACUACACAAAAGUACUG
control inhibitors	nucleotides	5'-CAGUACUUUUGUGUAGUACAAA
miR-148a-3p mimics	nucleotides	5'-UCAGUGCACUACAGAACUUUGU
miR-148a-3p inhibitors	nucleotides	5'-ACAAAGUUCUGUAGUGCACUGA

Note: F, Forward; R, Reverse.

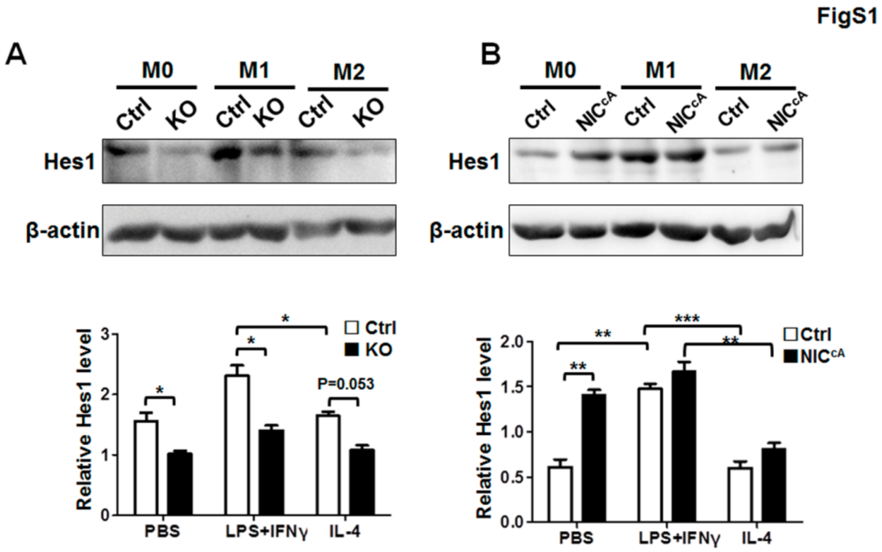

Figure S1. The efficiency of Notch knockdown or activation in macrophages was detected. (A) BMDMs from RBP-J^{cKO} or control (Ctrl) mice were stimulated with PBS (M0), LPS+IFN-γ (M1), or IL-4 (M2). The expression level of HES1, one downstream molecules of Notch signaling, was detected by Western blotting, using β-actin as a reference control and relative levels of HES1 compared quantitatively (n=3). (**B)** BMDMs were prepared from NIC^{cA} or control (Ctrl) mice, and stimulated with PBS (M0), LPS+IFN-γ (M1), or IL-4 (M2). The expression level of HES1 was detected by Western blotting, using β-actin as a reference control and relative levels of HES1 compared quantitatively (n=5).Bars, mean ± SD; $*P < 0.05$; $**P < 0.01$; $***P < 0.001$; n.s, not significant.

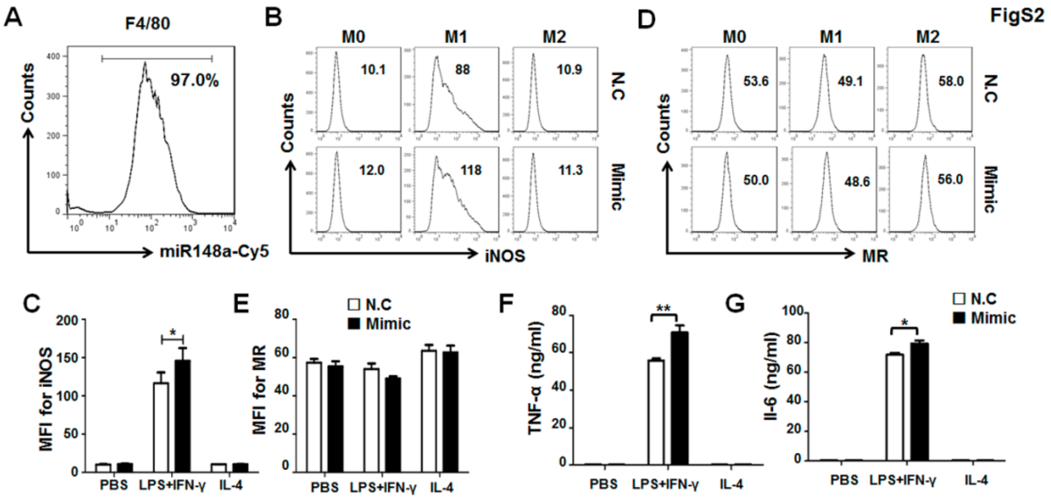

Figure S2. miR-148a-3p can promote M1 macrophage polarization. (**A)** The transfection efficiency of miR-148 in macrophages was detected by labeling miR-148a with Cy5 using FACS (n=3). (**B, C)** BMDMs were transfected with miR-148a-3p mimic or control (N.C). Cells were then polarized with PBS (M0), LPS+IFN-γ (M1), or IL-4 (M2) for 24 h. The expression level of M1 marker iNOS was analyzed using FACS intracellular staining (B), and then the mean fluorescent intensity (MFI) for iNOS was quantitatively compared (C)(n=3). (**D, E)** BMDMs were transfected with miR-148a-3p mimic or N.C. Cells were then polarized with PBS (M0), LPS+IFN-γ (M1), or IL-4 (M2) for 24 h. The expression level of M2 marker MR was analyzed using FACS intracellular staining **(D)**, and then the mean fluorescent intensity (MFI) for MR was quantitatively compared **(E)**(n=3). (**F, G)** BMDMs were transfected with miR-148a-3p mimic or N.C. Cells were then polarized with PBS (M0), LPS+IFN-γ (M1), or IL-4 (M2) for 24 h. After that, the supernatant of cultured medium was collected and the expression level of M1 marker TNF-α **(F)** and IL-6 **(G)** was analyzed using ELISA (n=8). Bars, mean ± SD; *P < 0.05; **P < 0.01.

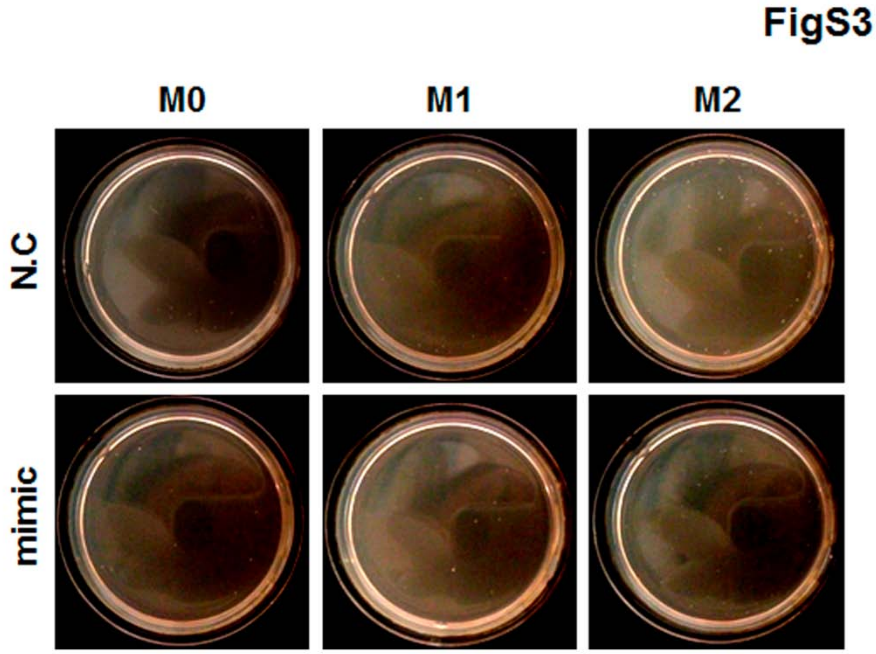

Figure S3. miR-148a-3p enhances bactericidal activity of macrophages. BMDMs derived from normal mice were transfected with miR-148a-3p mimic or control (N.C) and stimulated with PBS (M0), LPS+IFN-γ (M1), or IL-4 (M2). Then macrophages (1×10^6) were co-cultured with *E. coli* (1×10^7 CFU) that had been transformed with an *EGFP*-expressing vector for 2 h. Macrophages that had engulfed $EGFP^+$ bacteria were incubated for a further 6 h. Cells were then lysed and plated on ampicillin-containing agar plates to observe the growth of bacterial colonies (n=3).

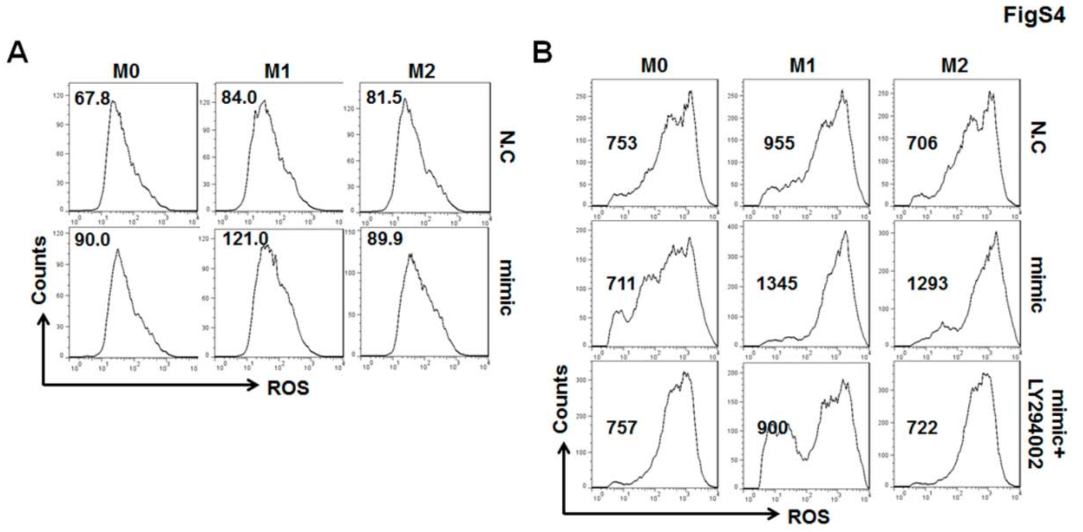

Figure S4. miR-148a-3p increases ROS level in macrophages in an AKT-dependent manner. (A) BMDMs were transfected with miR-148a-3p mimic or control (N.C), and then polarized with PBS (M0), LPS+IFN-γ (M1), or IL-4 (M2). ROS levels were determined by FACS (n=3). (**B)** BMDMs were transfected with miR-148a-3p mimic or control (N.C), and then polarized with PBS (M0), LPS+IFN-γ (M1), or IL-4 (M2), in the presence of LY294002. ROS levels were determined by FACS.

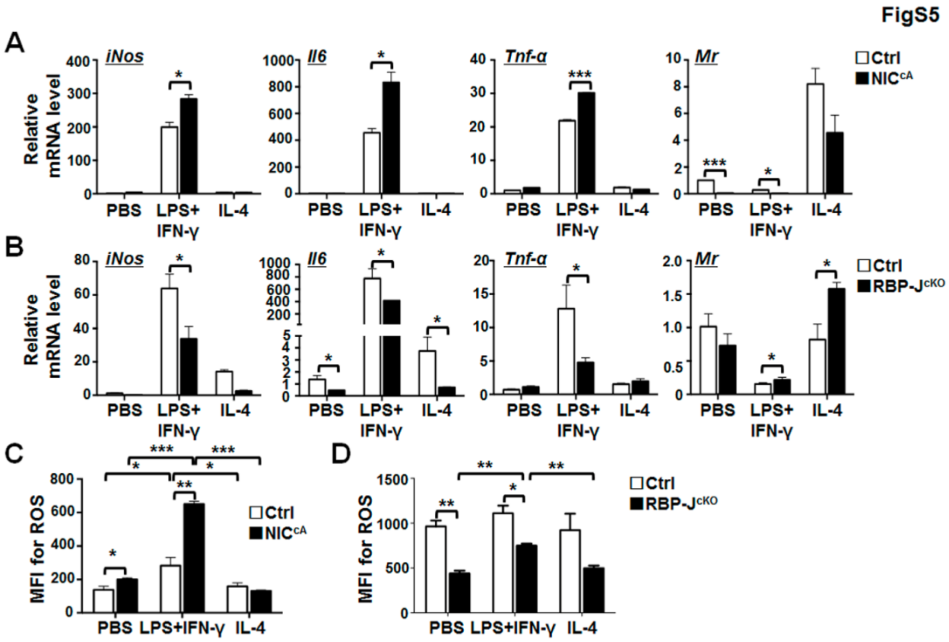

Figure S5. Notch signaling promotes M1 and attenuates M2 polarization of macrophages. (A, B) BMDMs from mice with different genotypes were stimulated with PBS (M0), LPS+IFN-γ (M1), or IL-4 (M2). mRNA levels of *iNos*, *Il6*, *Tnf-α*, and *Mr* were determined by qRT-PCR, using β-actin as a reference control (n=3). **(C, D)** BMDMs from mice with different genotypes were stimulated with PBS (M0), LPS+IFN-γ (M1), or IL-4 (M2). ROS levels were determined by FACS and quantified by MFI (n = 3). Bars, mean ± SD; *$P < 0.05$; **$P < 0.01$; ***$P < 0.001$.
